# Framing the future of the COVID-19 response operations in 2022 in the WHO African region

**DOI:** 10.1080/16549716.2022.2130528

**Published:** 2022-10-31

**Authors:** Thierno Balde, Boniface Oyugi, Humphrey Karamagi, Joseph Chukwudi Okeibunor, Ishata Nannie Conteh, Nonso Ephraim Ejiofor, Phionah Atuhebwe, Miriam Nanyunja, Amadou Bailo Diallo, Richard Mihigo, Zabulon Yoti, Fiona Braka, Abdou Salam Gueye

**Affiliations:** a World Health Organisation, Regional Office for Africa, Emergency Preparedness and Response Programme, Brazzaville, Congo; b Centre for Health Services Studies (CHSS), University of Kent, George Allen Wing, Canterbury UK; c Emergency Preparedness and Response Hub, World Health Organisation Emergency Hub for East and Southern Africa, Nairobi, Kenya; d World Health Organisation Emergency Hub for West and Central Africa, Dakar, Senegal

**Keywords:** COVID-19, response, operations, WHO African region

## Abstract

**Background:**

With the evolving epidemiological parameters of COVID-19 in Africa, the response actions and lessons learnt during the pandemic’s past two years, SARS-COV 2 will certainly continue to circulate in African countries in 2022 and beyond. As countries in the African continent need to be more prepared and plan to ‘live with the virus’ for the upcoming two years and after and at the same time mitigate risks by protecting the future most vulnerable and those responsible for maintaining essential services, WHO AFRO is anticipating four interim scenarios of the evolution of the pandemic in 2022 and beyond in the region.

**Objective:**

In preparation for the rollout of response actions given the predicted scenarios, WHO AFRO has identified ten strategic orientations and areas of focus for supporting member states and partners in responding to the COVID-19 pandemic in Africa in 2022 and beyond.

**Methods:**

WHO analysed trends of the transmissions since the first case in the African continent and reviewed lessons learnt over the past months.

**Results:**

Establishing a core and agile team solely dedicated to the COVID-19 response at the WHO AFRO, the emergency hubs, and WCOs will improve the effectiveness of the response and address identified challenges. The team will collaborate with the various clusters of the regional office, and other units and subunits in the WCOs supported with good epidemics intelligence. COVID-19 pandemic has afflicted global humanity at unprecedented levels.

**Conclusion:**

Two years later and while starting the third year of the COVID-19 response, we now need to change and adapt our strategies, tools and approaches in responding timely and effectively to the pandemic in Africa and save more lives.

## Introduction

**Figure 1.**
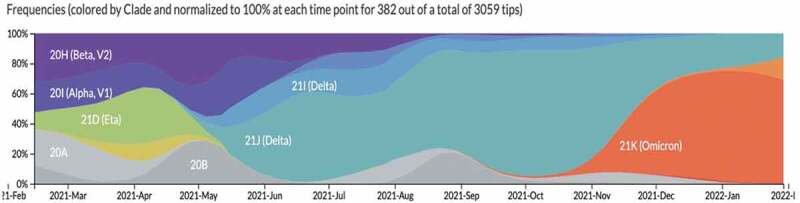
Virus evolution Genomic spread of SARS CoV 2 VOC.

Source: Nextstrain based on GISAID data: https://nextstrain.org/ncov/gisaid/global?f_region=Africa.

The African continent has been experiencing unprecedented health challenges due to the Coronavirus disease 2019 (COVID-19) pandemic [1], which have compounded the already difficult task the region was facing in moving towards Universal Health Coverage (UHC) attainment [2,3]. Twenty-four months into the pandemic since the first case of COVID-19 in the World Health Organization (WHO) African Region on 25 February 2020 into Algeria, all 47 African region countries have reported COVID-19 confirmed cases. As of 6 March 2022, 8,448,709 COVID-19 cases (accounting for 2% of total cases globally) and 170,300 deaths (accounting for 3% of total deaths globally) had been reported in WHO African region, with a cumulative case fatality rate (CFR) of 2.2% [4]. So far, the pandemic (in the WHO African region) has evolved into four main waves. The third (Delta) and the fourth (Omicron) waves have been characterised by: (i) difficulty in maintaining adherence to preventive measures resulting in clusters of cases in families, schools, workplaces and close settings [5,6]; (ii) the circulation of new variants of concern (VOCs), especially the Delta variant currently detected in all the countries in the WHO African region [7], and Omicron variant detected in 75% of the countries in the WHO African region (35 out of 47) (Figure 1); (iii) low testing capacities coupled with inappropriate testing strategies leading to under-detection of cases, particularly those with no or mild symptoms [8]. Equally, there have been variations in the estimations of cases and death across the continent [9].

The WHO assessment showed that only 14.2% – or one in seven – COVID-19 infections are being detected in Africa [10]. It is difficult to compare the number of cases and deaths experienced by the different countries for several reasons, such as being more or less likely to detect and report all COVID-19 cases and deaths; use of different case definitions and testing strategies or counting cases differently (for example, mild cases not being tested or counted); handling of time lags differently; differing quality of care or interventions being introduced at different stages of the illness; and the variation of the profiles of patients (for example, their age, sex, ethnicity and underlying comorbidities) between countries [11].

The introduction and spread of VOC are happening at different times and in different subregions [12–14]. The variants push many African countries to continue to battle the shutdown of health programmes and clinical services, pandemic fatigue, an exhausted workforce, and the consequences of the economic downturn. The latest pooled seroprevalence estimates^1^^1^Pooled estimates are based on national, sub-national and local studies from available countries (8 studies in 6 countries in September 2021). suggest that up to 65% of the population have some level of conferred immunity (as of September 2021) [15–17]. Current estimates of COVID-19 mortality are 618,000 deaths in the 18 months up to September 2021, but with associated reductions in mortality seen in upper and lower respiratory tract infections, road traffic accidents and diarrhoeal diseases, among others due to the response actions, such as public health and social measures out in place in response to the pandemic.^2^^2^Estimates provided from an ongoing modelling exercise by the WHO regional office for Africa data analytics teams. The fourth wave has seen numerous cases of alerts, resurgence, and situations of concern with all these factors. Eight African countries have already experienced/are currently experiencing a documented fourth wave [18].

In this paper, we highlight the progress of the different COVID-19 response pillars of the WHO Regional Office for Africa (AFRO). Using the progress, we situate the future of the COVID-19 response in Africa in 2022 and beyond using proposed interim scenarios of the evolution of the pandemic. With the proposed scenarios, we present the postulated strategic orientations (focus) of the COVID-19 response in Africa in 2022 and beyond that can guide the response of the member states in the region.

## Progress of the COVID-19 response in Africa

The dynamics of the response of COVID-19 in Africa has changed significantly over the past two years. The African region entered the pandemic with a strong position, buffered by tremendous experience resulting from previous health crises, such as polio, HIV, Ebola virus disease (EVD), and cholera [19,20]. Despite the health systems gap caused by inadequate health financing and the lack of sustained investment in healthcare [21,22], the continent has cast a wider net for vaccine procurement, began oxygen production and distribution on an industrial scale, increased intensive care capacity, consolidated community-based surveillance and case management standards, enhanced availability of trained healthcare workers (HCWs), and greatly improved laboratory and genomic testing [23].

Several strides have been made in boosting treatment capacity as the continent battles the pandemic and waves driven by more infectious variants. A scoping review of the preparedness, impact and response of the COVID-19 pandemic and healthcare systems in Africa showed that HCWs in the region have little knowledge of the detection and management of COVID-19 [24]. The HCWs were shown to have limited understanding of case definition, unable to identify high-risk patients, and conduct appropriate tests to identify suspected cases, lack adequate preventive measures to minimise transmission, and lack proper training. A survey conducted at the end of January 2020 to identify areas of potential weakness for COVID-19 preparedness and response among member states showed that patient treatment – also known as case management (CM) – was an area of particular concern for which the region was struggling [25]. To address the challenge, WHO and partners invested in training HCWs and 24 months into the pandemic had trained more than 12,000 medical officers and 44,000 nurses on COVID-19 treatment, surveillance standard operating procedures (SOPs), tools and guidelines across the region [26]. With the constant changing treatment protocols, many countries constantly updated their national treatment and therapeutics guidelines to include novel recommended COVID-19 therapeutics that arose as research developed in that area with the support of partners, as shown from the thematic analysis of WHO intra-action review reports [27]. Over the past two years, international emergency medical teams (EMTs) have been deployed to 25 countries where more than 6,000 HCWs have been trained [26]. The clinical CM capacities have been enhanced in that the number of Intensive Care Unit (ICU) beds has increased across the African continent, from 8 per 1 million people in 2020 to 20 per 1 million today; while that of oxygen production plants has increased from 68 to 363 through the repair, maintenance and procurement of new oxygen plants [28]. Where plants have been set up, the cost of oxygen has decreased by 40% [28].

The testing capacities improved. At the onset of the pandemic, only two countries in the region (South Africa through National Institute for Communicable Diseases (NICD) and Senegal through Institut Pasteur Dakar (IP-D)) had the capacity to test for the virus and two years later, all countries and over 900 laboratories can now detect the virus using the standard polymerase-chain reaction method [26]. Testing was further boosted with antigen rapid diagnostic tests (Ag-RDT) introduced in the region in October 2020 [29,30]. As of 28 February 2022, more than 93 million tests had been carried out through PCR and Ag-RDT. Additionally, on 10 September 2020, the WHO in collaboration with the Africa Centres for Disease Control and Prevention (A-CDC) launched a network of 12^3^^3^Kwazulu-Natal Research Innovation and Sequencing Platform (KRISP), African Center of Excellence for Genomics of Infectious Diseases (ACEGID), South African National Bioinformatics Institute (SANBI), National Institute for Communicable Diseases (NICD), Kenya Medical research Institute (KEMRI), Uganda Virus Research Institute (UVRI), Institut Pasteur Dakar (IPD), Noguchi Memorial Institute for Medical Research (NMIMR), Institut National pour la Recherche Biomedicale (INRB), Nigeria Centre for Disease Control (NCDC) and Centre International de Recherches Medicales de Franceville in Gabon (CIRMF). genomic surveillance laboratories to track, identify, and bolster response to COVID-19 variants and other emerging pathogens. The network has produced more than 90,000 genomes – above 50,000 targets for 2021 since close collaboration with the network and governments [26]. Through partnership with the South African National Bioinformatics Institute (SANBI), WHO has established a Regional Centre of Excellence for Genomic Surveillance and Bioinformatics in Cape Town, to initially support 14 southern countries, increasing their sequencing capacity by five-fold monthly before expanding to serve more countries [31]. The centre provides sequencing, data analysis and other technical support services for national activities, neighbouring countries and countries in their sub-regions in a timely manner and has helped countries detect the presence and understand the impact of VOCs, notably the Delta variant (ibid).

To date, 28 countries members of the African Regional Influenza Laboratory network are not only implementing sentinel surveillance for influenza-like illness (ILI) and/or severe acute respiratory infection (SARI) using WHO standards but also contributing to weekly influenza surveillance reporting at the regional and global levels. Despite significant progress in the African region on establishing sentinel influenza surveillance in the countries, there is still a paucity of epidemiological and virological data as not all countries have sentinel influenza surveillance.

The pandemic brought attention to the disparities in the essential medical supplies and equipment for diagnostics. As of the writing of this paper, 6,671 oxygen concentrators, more than 400 ventilators, 1,516 patient monitors, and 15,656 pulse oximeters have been shipped to 47 Member States. Additionally, about 105.6 million items of personal protective equipment (PPE), 70.4 million laboratory tests and reagents, including 50.4 million Ag-RDT kits and 7.4 million sample collection kits, have been delivered to countries on the Continent [26].

Over the past two years, the adherence to preventive measures and acceptance of COVID-19 vaccine using up to 5,000 national experts trained on the key areas of community engagement, including coordination with partner organisations and addressing misinformation and rumours, has been promoted [26]. As at the writing of this paper, there are 27 countries currently being supported to carry out knowledge, attitude, and practice studies to better understand and respond to community concerns [26] and whose results will be availed at the tail end of the research. Countries have also reinforced collaboration with national media, health promotion teams and religious leaders to enhance community involvement in COVID-19 prevention and response.

Some eight countries^4^^4^Burundi, Congo, Cote d’Ivoire, DRC, Guinea-Bissau, Mozambique, Senegal, Zambia. are currently piloting community-based surveillance to scale up these cost-effective Ag-RDTs, which helps augment COVID-19 case detection and management in the communities. The programme aims to reach more than 7 million people with Ag-RDT in the next year and increase testing capacity in participating countries by 40%. Early results from hotspot districts in six countries show that 20,584 Ag-RDT have been performed so far, contributing to more than half of all tests in these areas and leading to an additional 364 new cases detected (i.e. 10% of all cases reported from these areas) [32]. Additionally, early results from three countries also showed that compliance to public health and safety measures in selected hotspot communities improved to 35% on a second assessment, up from 18% during the first assessment (ibid).

Correspondingly, there is a scaling-up of research activities, notably through some seroprevalence studies (that directly measure antibodies, acquired by either natural infection or vaccination, at a population level, providing a profile of humoral immunity within a population) [33,34] and operational research studies to better understand the dynamics of the COVID-19 pandemic within the African region and generate evidence to inform operational planning and understand vaccine effectiveness. The designs of the studies incorporated include: cross-sectional and repeat cross-sectional surveys, and prospective and retrospective cohort studies [35]. Overall, the current COVID-19 surveillance and reporting suggest greater population exposure to SARS-CoV-2 and protection against COVID-19 disease than indicated by surveillance data. There is an estimated infection to confirmed case ratio of 97:1 in the continent, ranging from 10:1 to 958:1 across countries. The seroprevalence is high but also differs both within countries – urban vs rural (lower seroprevalence for rural geographic areas), children vs adults (children aged 0–9 years had the lowest seroprevalence) - and between countries and African sub-regions (Eastern, Western, and Middle African regions associated with higher seroprevalence) [35].

The vaccination component was added to the COVID-19 response package to increase immunity rapidly, but its rollout has been slower in most African countries than in other parts of the world. For instance, as of the writing of this paper, the African Region of the WHO has received 677,800,349 doses of vaccines since the first shipment in late February 2021, of which 435,069,897 (64.2%) were from the COVID-19 Vaccines Global Access (COVAX) facility which is the vaccines pillar of the Access to COVID-19 Tools (ACT) Accelerator while 200,567,802 (29.6%) was secured through bilateral agreements, and 42,162,650 (6.2%) was through the African Vaccine Acquisition Trust (AVAT) which acts as a centralised purchasing agent on behalf of the African Union (AU) Member States.^5^^5^Detailed discussion on the Joint Statement on Dose Donations of COVID-19 Vaccines to African Countries is found here: https://www.who.int/news/item/29-11-2021-joint-statement-on-dose-donations-of-covid-19-vaccines-to-african-countries. As of 7 March 2022, in the African region, a total of 164,727,289 persons (11.9% of the population) were fully vaccinated [36]. Seven countries have already surpassed 40% of the people fully vaccinated (Seychelles 80.5%, Mauritius 74.4%, Morocco 62.1%, Rwanda 56.7%, Tunisia 52.6%, Cape Verde 52.0%, Botswana 46.9% (ibid)).

The IPC components have also improved significantly through the capacity building and guidelines on IPC interventions in the context of COVID-19 resurgence targeting all 47 countries. The training components have supported best practices implementation of IPC measures in the health facilities and the community using the IPC scorecard (a scorecard adapted from IPC work on Ebola response for the rapid evaluation of health facilities on the availability of parameters such as: IPC coordinator or IPC team, isolation areas, triage, hand hygiene stations, security of patients and families, adequate number of HCW, PPE availability, PPE utilisation, waste management, and HCW trained in IPC [37]). Additionally, they have focused on best practices on PPE supply management and improving vaccination compliance in HCWs. Further, a regular assessment of IPC in the community has been established in line with local epidemiology besides the monitoring and supervision visits.

Additionally, WHO regional office for Africa (AFRO) is working with partners (such as Africa Centres for Disease Control and Prevention, United Nations International Children’s Emergency Fund (UNICEF), Gavi, the Vaccine Alliance, and fact-checking media organisations) in Africa Infodemic Response Alliance (AIRA), a unique and independent platform and network for sharing science-based facts on health and countering misinformation [38]. It has helped countries set up platforms to apply infodemic response methods developed by over 1300 experts from various disciplines and provides support to the region and targeted support to eight priority countries.^6^^6^Angola, the Democratic Republic of the Congo, Guinea, Kenya, Mali, Niger, Nigeria and South Africa.

## The future of the COVID-19 response in Africa in 2022 and beyond

The Incident Management Support Team (IMST) structure has been premised on the Emergency Response Framework (ERF) [39] guidance which is applicable for all graded emergencies but inadequate for a pandemic where all the countries need support at the same time. Currently, the response uses a strategic approach where staff are mobilised and repurposed from all clusters to help meet the increased demand. There has been timely support to countries to operationalise the management structure for the in-country response as defined in the country plans. The IMST preparedness structure at WHO AFRO was rapidly transformed into a COVID-19 IMST response structure, following the first few cases reported. It facilitated early response in all 47 Member States. In most countries, the incident management team (IMT) established at the WHO Country Office (WCO) was used as an example to set up a ministry of health (MoH) incident management structure/emergency operation Centres (EOC).

Considering the epidemiological evolution of the COVID-19 in Africa, the response actions, accomplishments, and lessons learnt during the past one, and a half year, it is certain that the SARS-COV 2 will continue to circulate in African countries in 2022 and beyond. In this regard, countries in the African continent need to be more prepared and plan to *‘live with the virus’* for the upcoming two years and after; and at the same time mitigate risks by protecting the most vulnerable and those responsible for maintaining essential services. From the literature review and previous modelling exercises conducted globally and regionally [40–43], three new factors might impact the evolution, dynamic and intensity of the COVID-19 pandemic in Africa in 2022 and beyond: i) the risk of re-infection of people who have already been affected by the COVID-19 [44,45]; ii) the level of acquired immunity of the population either through natural infection and the vaccination [46,47]; and iii) the occurrence of new variants of the SARS-COV 2 with higher transmissibility, virulence and/or capacity to escape immunity induced by prior infection or vaccination [48,49]. These factors and the evolution are not specific to Africa but the globe; however, the African continent is of concern because of the traditionally weak health care system.

The complex interplay and the dynamic change of these factors indicate that the strategy by the region should be agile and multi-layered. Therefore, for planning purposes, the WHO AFRO is anticipating four interim scenarios of the evolution of the pandemic in 2022 and beyond in the region (Box 1).

**Table:** 

**Box 1: Scenarios** Scenario 1: continuous trend with similar virus and response actions (status quo), with no re-infection – Optimistic Scenario but highly unlikely Scenario 2: continuous trend with similar virus and response actions but varied effects of immunisation on the number of cases and deaths Scenario 3: Upsurge of new cases due to a high level of re-infection Scenario 4: A new VOC with higher transmissibility and/or virulence (worst-case scenario)

The detailed methodological approach for estimating the scenario is presented elsewhere [50,51]. It is built on the existing models explaining the past and present and predicting the future of the SARS-CoV-2 virus in the WHO African region countries. The modelling utilised the framework Partially Observed Markov Process (POMP) models to track the population in the susceptible (S), exposed (E), infected (I), recovered (R) and dead (D) compartments of the model. A detailed definition of each component is published elsewhere [51].

The modelling used historical data on new cases, deaths and vaccination obtained from the WHO Coronavirus (COVID-19) dashboard. At the same time, data on social behaviour, ecological, morbidity and the virus characteristics assumptions were gathered from a meta-analysis of existing literature. The modelling incorporates parameters and assumptions of the unique virus characteristics per country (transmissibility: driven by attack rate, and country-specific socioecological factors (population density, population mobility, personal hygiene safety)); re-infection rate (different for vaccination (9 months) and natural immunity (6 months)); and severity of disease (driven by burden of hypertension, diabetes mellitus, cardiovascular diseases, chronic obstructive pulmonary diseases (COPD), physical inactivity, age, HIV burden); omicron effect (reduce re-infection rate (estimate of 20% higher re-infection)); current dynamics of the vaccination; VOCs each have unique characteristics (new epidemics); immunity that leads to a 90% reduction in severity/death for future re-infections; the future patterns are informed by past events; and many cases and deaths are not reported/hidden (due to multiple reasons). The combination of these new factors with other pre-existing ones (such as the intensity and quality of the response, increasing ‘fatigue’ of the population in adhering to public and response measures, low rates of vaccination, and weak health systems) will determine the quality and the impact of the future response to the pandemic.

The model was constructed both in Microsoft Excel and R software (Version 4.1.2) for internal validation. Both were set up to run weekly from WHO – Dynamic Integrated Modelling Systems for COVID-19 in the African Region: Past, Present and Future [51].

### Scenario 1: continuous trend with similar virus and response actions (status quo), with no re-infection – optimistic scenario but highly unlikely

At the current level of engagement of communities in adhering to public health response measures, the current status of COVID-19 re-infection for each of the countries in the region, and slow progress of vaccination, the pandemic is predicted to continue to spread in African countries at a moderate level with the presence of upsurges during some specific periods (such as cold/winter seasons, festive period, specific events with mass gathering such as elections, and major sportive events) as shown in Figure 1. However, it is unlikely the peaks seen in the past two years would recur. Additionally, mortality would be lower than has been experienced so far because the most vulnerable groups were prioritised for vaccination in the early phases of the rollout. Given that evidence has shown that scale-up of vaccination did not prevent subsequent waves of infection but did reduce deaths and hospitalisation [52]; in this scenario, the WHO will work with the member states to enhance community and hospital surveillance and response capacities during low-level transmission periods and respond timely and effectively during the upsurge of cases.

### Scenario 2: continuous trend with similar virus and response actions but varied effects of immunisation on the number of cases and deaths

**Figure 2.**
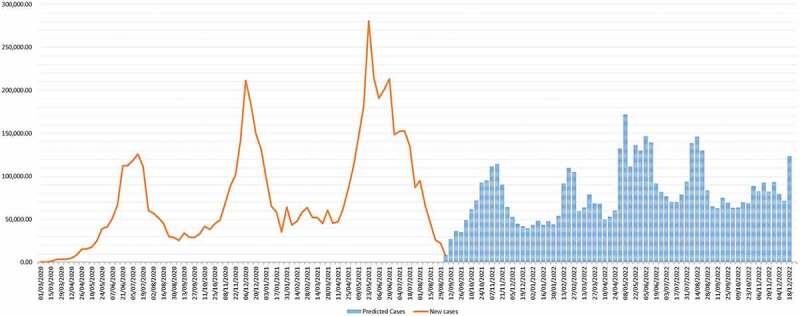
Show predicted scenario 1 at the current level of adhesion to public health measures, rates of contacts, re-infection, variants and immunisation levels; the disease is predicted to remain in the community, though with lower peaks in 2022.

**Figure 3.**
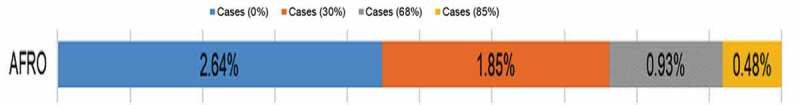
Cases as a percentage of the population at different levels of immunisations at the end of 2022.

This scenario envisages the continuous trend with similar virus and response actions (status quo) but the various status of immunisation (along 0, 30, 68 and 85%) as shown in Figures 2 and 3. If immunisation were at 0%, then 2.64% of the population would be infected in the AFRO region by the end of 2022. These proportions would change to 1.85, 0.93, and 0.48% for 30, 68, and 85% immunisation levels, respectively (Figure 2). The percentage reduction of mortality would be proportional to the various immunisation levels, with variations from country to country, as shown in Figure 3. Detailed analysis per country is in Appendix 1, and the assumptions of the detailed assumptions of the immunisation levels on deaths and cases are discussed elsewhere [50,51]. The situation varies across the different countries due to their inherent characteristics and the variants of the virus therein. In this scenario, the WHO will work with the member states to scale up vaccination, maintain surveillance and contact tracing and testing, and support for review response plan and contingency for upsurge cases.

### Scenario 3: upsurge of new cases due to a high level of re-infection

**Figure 4.**
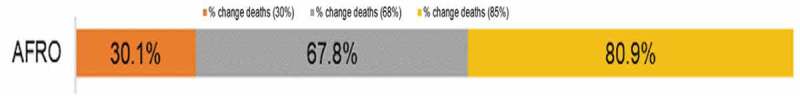
Percentage reduction of deaths among the reported cases with varying levels of immunisations.

**Figure 5.**
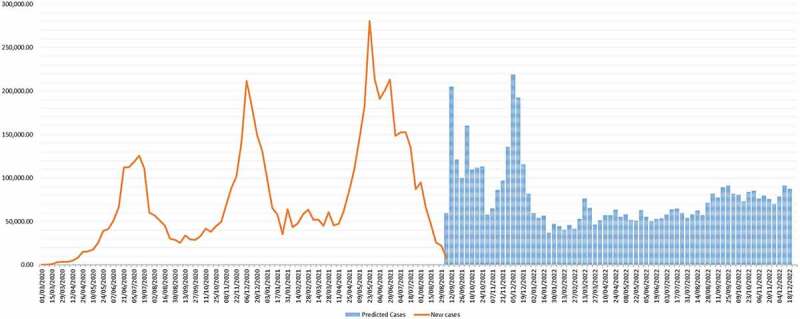
Predicted scenario 2 with risk of re-infection at 40%.

The average level of re-infection (within one year) in the African region varies from 5 to 20%, given the current rate of VOCs, vaccinations, and other parameters described above. However, countries like Seychelles have experienced the highest re-infection rate at 60%, while South Africa has had a 40% re-infection rate. A 40 or 80% re-infection rate implies an important changing dynamic of the pandemic in Africa with higher peaks in 2022, as shown in Figures 4 and 5.

In this scenario, WHO AFRO will support member states to develop and implement strategies for scaling community-based response actions to limit the transmission in high-risk localities (breaking the chains of community transmission) and prevent the importation of cases. It is important to realise that certain measures, such as wearing masks in crowded, poorly ventilated spaces and physical distancing, will need to be enhanced even among those vaccinated, particularly to protect vulnerable groups. Additionally, vaccination strategies, including additional or booster doses, depending on who is getting re-infected and how the use of additional and booster doses will be prioritised in supply-constrained situations.

### Scenario 4: a new VOC with higher transmissibility and/or virulence (worst-case scenario)

**Figure 6.**
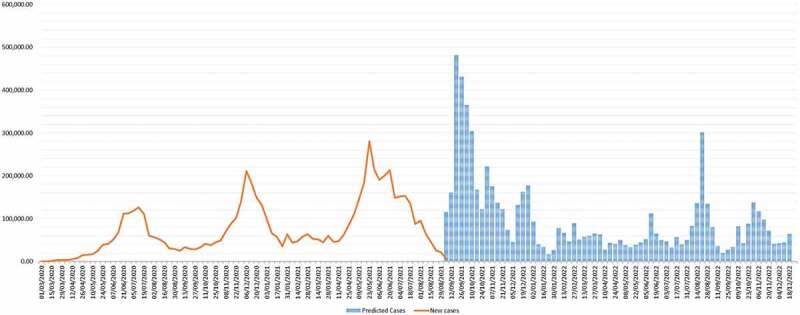
Predicted scenario 2 with risk of re-infection at 80%.

Given the continuous circulation of the virus, there is a risk of mutation of the virus with the occurrence of new, more transmissible and/or virulent variants, which can lead to severe diseases and deaths. More variants affecting transmissibility are more likely, and the resultant scenario is as shown in Figure 6.

**Figure 7.**
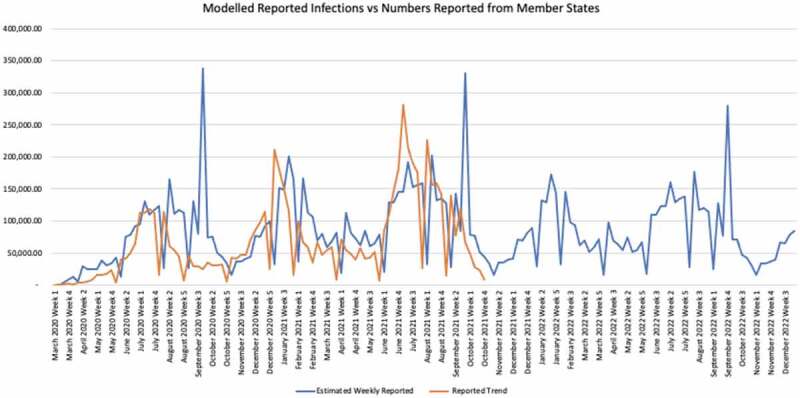
Predicted scenario 3 with the introduction of a new variant.

In this scenario, more resources and efforts need to be mobilised by the WHO to support member states to respond by limiting the transmission of the disease, increasing the treatment capacities, and scaling up the genomic surveillance of the variants. Also, the vaccination strategies will need to be revised, depending on the virulence of the variant, those most affected and the immune escape potential of the variant. Figure 7

## Main strategic orientations of the COVID-19 response in Africa in 2022 and beyond

The overall objectives of the WHO/AFRO EPR department are to help *‘reduce the health consequences of public health emergencies … by effective national and international risk reduction, preparedness, alert, response and recovery actions.’* [53] The last one and a half years have seen intensive response operations in the African region under the IMST with core emergency response activities, including the deployment of critical experts in all the different response pillars, mainly to support coordination, surveillance, IPC, laboratory, CM, and Risk Communication and Community Engagement (RCCE), Vaccine deployment, in addition to supplies and equipment and provision of guidance documents.

In preparation for the rolling out of response actions given the predicted scenarios mentioned above, and based on analysis and lessons learnt over the past months, WHO AFRO has identified the following strategic orientations and areas of focus for supporting member states and partners in responding timely and effectively to the COVID-19 pandemic in Africa in 2022 and beyond:

**Table:** 

**Box 2: Main Strategic orientations of the COVID-19 response in Africa in 2022 and beyond** Reinforcing COVID-19 surveillance capacities (through multisource surveillance systems such as indicator-based and event-based surveillance for hospital, primary healthcare and community level, hospital surveillance for new cases and essential services implications, using II and SARI sentinel surveillance) and scaling up COVID-19 testing and genomic/variant surveillance capacities. Defining and implementing adapted community-based response actions Improving the CM capacities through increasing oxygen and treatment capacities from the health system perspective Increasing the vaccination uptake through community engagement, advocacy and ownership and updating vaccination strategies and targets, based on the evolving epidemiological scenario and on emerging evidence on the performance of vaccines, effectiveness against different variants Reinforcing COVID-19 M&E, data and intelligence collection and use for orienting and guiding response actions Maintaining and reinforcing critical human resource capacities in WCOs and member states countries to respond to upsurge of COVID-19 cases Augment the medical and non-medical supplies and other material and equipment for addressing timely operational needs required by member states countries Reinforce and diversify the collaboration and coordination with existing and new partners (academia, regional economic and political entities, CSOs, Private sector organisations) Reinforcing and scaling-up fundamental and operational research to guide response actions, Transitioning progressively COVID-19 response capacities into the formal health system.

## Conclusion and looking ahead

Two years into the COVID-19 response based on the Incident Management System (IMS) principles and approaches, and given feedback and learnings obtained from the ongoing intra-action review (IAR) of the IMST, it is imperative to define a more adapted and agile response structure that can manage the future COVID-19 response. Additionally, establishing a core and agile team solely dedicated to the COVID-19 response at the WHO AFRO, the emergency hubs, and WCOs will improve the effectiveness of the response and address identified challenges. The team will collaborate with the various clusters of the regional office, and other units and sub-units in the WCOs supported with good epidemics intelligence. COVID-19 pandemic has afflicted global humanity at unprecedented levels. Two years after and while starting the third year of the COVID-19 response, we now need to change and adapt our strategies, tools and approaches in responding timely and effectively to the pandemic in Africa and save more lives.

## Paper context

The paper positions the countries in Africa to be more prepared and plan to ‘live with the COVID-19 virus.’ The evolution of the pandemic in 2022 and beyond in the region envisages scenarios to guide response actions. There is a need to mitigate risks by protecting the most vulnerable and those responsible for maintaining essential services. Additionally, it is imperative to change and adapt our strategies, tools and approaches in responding timely and effectively to the pandemic in Africa and save more lives.

## Appendix 1

a) Cases as a percentage of the population at different levels of immunisations at the end of 2022

b) Percentage reduction of deaths among the reported cases with varying levels of immunisation
